# Supplementary material for: Antimicrobial Susceptibility of Fresh Produce-Associated Enterobacteriaceae and Enterococci in Oman
Source: Foods. 2022 Oct 5;11(19):3085. doi: 10.3390/foods11193085 (PMC9562674; doi:10.3390/foods11193085)
Supplement: Supplementary file 1 [file foods-11-03085-s001.zip › foods-1934768-supplementary.pdf]

Table S1. Type of antibiotics and the interpretive criteria (CLSI, 2015) for inhibition zone diameters (mm) of various antibiotics and for vancomycin Minimum Inhibitory Concentration; MIC (μg/mL) used for enterococci.

Antibiotic	Interpretive criteria		
	Susceptible	Intermediate	Resistant
Ampicillin (AMP 10 μg)	≥ 17	-	≤ 16
Ciprofloxacin (CIP 5 μg)	≥ 18	13-17	≤ 12
Chloramphenicol (C 30 μg)	≥ 18	13-17	≤ 12
Erythromycin (E 15 μg)	≥ 23	14-22	≤ 13
Gentamicin (GM 500 μg/mL)	No growth	-	Growth of >1 colony
Nitrocefin	No color change	-	Color change to red
Penicillin G (10 μg)	≥ 15	-	≤ 14
Streptomycin (S 2000 μg/mL)	No growth	-	Growth of >1 colony
Tetracycline (TE 30 μg)	≥ 19	15-18	≤ 14
Vancomycin (VA 256-0.015 μg/mL)	≤4	8-16	≥ 32

-Interpretive criteria are not set.

Table S2. Chlorhexidine MIC of Enterobacteriaceae and enterococci isolated from local and imported fresh produce and the bacterial Antibiotic Resistance Index (ARI).

Bacteria No.	Bacteria identity (PCR)	Source	MIC (µg/mL)	ARI
2	*Escherichia coli*	Cabbage, Oman	2	0.14
3	*Escherichia coli*	Cabbage, Oman	1	0.00
4	*Escherichia coli*	Lettuce, Jordan	2	0.21
5	*Escherichia coli*	Lettuce, Jordan	1	0.29
6	*Escherichia coli*	Radish, Oman	1	0.07
7	*Escherichia coli*	Radish, China	2	0.21
8	*Escherichia coli*	Cabbage, Oman	2	0.36
11	*Escherichia coli*	Lettuce, Jordan	1	0.36
12	*Escherichia coli*	Radish, Oman	2	0.00
14	*Escherichia coli*	Radish, Oman	2	0.07
15	*Escherichia coli*	Radish, China	1	0.21
19	*Enterococcus faecium*	Cabbage, Oman	2	0.10
20	*Enterococcus mundtii*	Cabbage, Oman	2	0.00
21	*Enterococcus sulfureus*	Cabbage, Oman	2	0.00
22	*Enterococcus casseliflavus*	Cabbage, Netherlands	4	0.20
23	*Enterococcus casseliflavus*	Cabbage, Netherlands	2	0.20
24	*Enterococcus casseliflavus*	Cabbage, Netherlands	4	0.30
25	*Enterococcus casseliflavus*	Cucumber, UAE	4	0.20
26	*Enterococcus faecalis*	Lettuce, Jordan	8	0.30
27	*Enterococcus sulfureus*	Lettuce, Jordan	2	0.30
28	*Enterococcus faecium*	Lettuce, Jordan	2	0.10
29	*Enterococcus faecalis*	Lettuce, Iran	8	0.30
30	*Enterococcus casseliflavus*	Lettuce, Iran	2	0.20
31	*Enterococcus mundtii*	Lettuce, Iran	2	0.00
32	*Enterococcus faecalis*	Radish, Oman	8	0.20
33	*Enterococcus mundtii*	Radish, Oman	2	0.00
34	*Enterococcus faecalis*	Radish, Oman	4	0.20
35	*Enterococcus casseliflavus*	Radish, China	2	0.30
36	*Enterococcus mundtii*	Radish, China	2	0.20
37	*Enterococcus casseliflavus*	Radish, China	4	0.10
38	*Enterococcus raffinosus*	Dates, Oman	1	0.10
39	*Enterococcus casseliflavus*	Dates, Saudi Arabia	4	0.30
40	*Enterococcus faecalis*	Mango, Oman	4	0.10
41	*Enterococcus faecalis*	Papaya, Oman	4	0.20
42	*Enterococcus faecalis*	Papaya, Oman	4	0.20
43	*Enterococcus hirae*	Papaya, Oman	4	0.00
44	*Enterococcus casseliflavus*	Tomato, Syria	2	0.10
45	*Enterococcus gilvus*	Tomato, Syria	4	0.00
46	*Enterococcus casseliflavus*	Watermelon, Oman	4	0.20
47	*Enterococcus casseliflavus*	Watermelon, Iran	4	0.10

48	*Enterococcus casseliflavus*	Watermelon, Iran	4	0.20
49	*Enterococcus casseliflavus*	Watermelon, Iran	4	0.10
50	*Klebsiella pneumoniae*	Cabbage, Oman	16	0.07
51	*Klebsiella pneumoniae*	Cabbage, Oman	8	0.07
52	*Enterobacter cloacae*	Cabbage, Oman	16	0.36
53	*Raoultella planticola*	Cabbage, Netherlands	64	0.07
54	*Kluyvera intermedia*	Cabbage, Netherlands	4	0.29
55	*Pantoea agglomerans*	Cabbage, Netherlands	2	0.07
56	*Enterobacter asburiae*	Carrot, Oman	4	0.36
57	*Enterobacter cloacae*	Carrot, Oman	8	0.29
58	*Klebsiella oxytoca*	Carrot, Oman	16	0.14
59	*Rahnella aquatilis*	Carrot, Australia	2	0.14
60	*Pantoea agglomerans*	Carrot, Australia	1	0.00
61	*Rahnella aquatilis*	Carrot, Australia	2	0.14
62	*Serratia liquefaciens*	Carrot, USA	4	0.14
63	*Enterobacter amnigenus*	Carrot, USA	2	0.07
64	*Pantoea agglomerans*	Carrot, USA	2	0.07
65	*Pantoea agglomerans*	Capsicum, Oman	1	0.29
66	*Pantoea agglomerans*	Capsicum, Oman	1	0.29
67	*Erwinia aphidicola*	Capsicum, Jordan	2	0.21
68	*Klebsiella pneumoniae*	Capsicum, Jordan	8	0.07
69	*Erwinia aphidicola*	Capsicum, Jordan	2	0.14
70	*Pantoea agglomerans*	Capsicum, UAE	1	0.00
71	*Enterobacter ludwigii*	Capsicum, UAE	4	0.29
72	*Pantoea dispersa*	Cucumber, Oman	2	0.14
73	*Pantoea dispersa*	Cucumber, Oman	2	0.07
74	*Pantoea dispersa*	Cucumber, Oman	2	0.14
75	*Enterobacter ludwigii*	Cucumber, UAE	16	0.36
76	*Erwinia aphidicola*	Cucumber, UAE	2	0.07
77	*Erwinia rhapontici*	Cucumber, UAE	2	0.14
78	*Enterobacter amnigenus*	Lettuce, Oman	4	0.07
79	*Enterobacter amnigenus*	Lettuce, Oman	2	0.07
80	*Enterobacter cancerogenus*	Lettuce, Oman	4	0.21
81	*Pantoea agglomerans*	Lettuce, Jordan	2	0.07
82	*Rahnella aquatilis*	Lettuce, Iran	2	0.14
83	*Enterobacter cloacae*	Lettuce, Iran	8	0.29
84	*Enterobacter cloacae*	Lettuce, Iran	32	0.36
85	*Pectobacterium carotovorum*	Radish, Oman	4	0.00
86	*Enterobacter cloacae*	Radish, Oman	8	0.29
87	*Enterobacter cloacae*	Radish, Oman	16	0.07
88	*Klebsiella oxytoca*	Radish, China	16	0.07
89	*Pantoea agglomerans*	Radish, China	1	0.00
90	*Enterobacter amnigenus*	Radish, China	8	0.00
91	*Pantoea dispersa*	Banana, Oman	2	0.36

92	*Pantoea cypripedii*	Banana, Oman	2	0.00
93	*Klebsiella pneumoniae*	Banana, Philippines	16	0.07
94	*Klebsiella pneumoniae*	Banana, Philippines	32	0.07
95	*Klebsiella pneumoniae*	Dates, Oman	8	0.07
96	*Klebsiella pneumoniae*	Dates, Oman	16	0.07
97	*Klebsiella pneumoniae*	Dates, Oman	16	0.07
98	*Enterobacter oryzae*	Dates, Saudi Arabia	2	0.07
99	*Pantoea agglomerans*	Dates, Saudi Arabia	2	0.00
100	*Pseudocitrobacter faecalis*	Mango, Oman	16	0.21
101	*Klebsiella pneumoniae*	Mango, Oman	16	0.07
102	*Klebsiella pneumoniae*	Mango, Oman	16	0.07
103	*Pantoea dispersa*	Mango, India	1	0.21
104	*Pantoea dispersa*	Mango, Pakistan	1	0.07
105	*Enterobacter cloacae*	Papaya, Oman	8	0.29
106	*Klebsiella pneumoniae*	Papaya, Oman	16	0.07
107	*Enterobacter cloacae*	Papaya, Oman	16	0.21
108	*Pantoea eucrina*	Papaya, Thailand	1	0.14
109	*Pantoea dispersa*	Papaya, Thailand	2	0.14
110	*Enterobacter cloacae*	Papaya, Thailand	16	0.00
111	*Enterobacter cloacae*	Papaya, Philippines	4	0.29
112	*Escherichia hermannii*	Papaya, Philippines	32	0.07
113	*Enterobacter cloacae*	Papaya, Philippines	16	0.07
114	*Serratia marcescens*	Tomato, Oman	8	0.36
115	*Klebsiella oxytoca*	Tomato, Oman	32	0.07
116	*Enterobacter ludwigii*	Tomato, Oman	16	0.29
117	*Citrobacter freundii*	Tomato, Netherlands	16	0.36
118	*Pantoea vagans*	Tomato, Syria	2	0.07
119	*Enterobacter cloacae*	Tomato, Syria	32	0.29
120	*Enterobacter hormaechei*	Tomato, Syria	32	0.29
121	*Enterobacter cloacae*	Watermelon, Oman	32	0.29
122	*Enterobacter cloacae*	Watermelon, Egypt	32	0.29
123	*Enterobacter cloacae*	Watermelon, Egypt	32	0.29
124	*Klebsiella pneumoniae*	Watermelon, Iran	8	0.07
125	*Pantoea agglomerans*	Watermelon, Iran	2	0.00
126	*Klebsiella pneumoniae*	Watermelon, Iran	16	0.07
129	*Escherichia coli*	ATCC 25922	2	0.00
130	*Enterococcus faecalis*	ATCC 29212	4	0.20
131	*Enterococcus faecalis*	ATCC 51299	8	0.30
132	*Klebsiella pneumoniae*	ATCC BAA-1705	16	0.93

Table S3. Mean diameter (mm) of growth inhibition zones of 6 antibiotics, MIC of vancomycin and susceptibility to high level gentamicin (GM), nitrocefin (N) and high level streptomycin (S) of enterococci isolated from local (n $_{bacteria}$ = 12) and imported (n $_{bacteria}$ = 19) fresh produce.

Bacteria No.	Amp	CIP	C	E	GM	N	P	S	TE	VA
19	26	20	23	26	NG	NCC	21	NG	26	0.25
20	27	22	24	27	NG	NCC	22	NG	27	0.25
21	31	23	25	28	NG	NCC	29	NG	28	0.5
22	27	17	24	17	NG	NCC	26	NG	28	2
23	34	18	24	18	NG	NCC	33	NG	30	4
24	25	19	23	20	NG	NCC	23	NG	26	8
25	29	22	27	17	NG	NCC	29	NG	9	4
26	22	19	22	20	NG	NCC	20	NG	8	1
27	30	22	11	7	NG	NCC	22	NG	6	0.5
28	27	22	23	22	NG	NCC	22	NG	26	0.5
29	27	19	21	19	NG	NCC	21	NG	7	1
30	29	20	22	19	NG	NCC	29	NG	28	4
31	26	22	22	24	NG	NCC	21	NG	27	0.5
32	26	19	21	22	NG	NCC	21	NG	26	4
33	27	25	23	31	NG	NCC	19	NG	30	0.5
34	27	19	22	22	NG	NCC	21	NG	25	2
35	24	20	20	6	NG	NCC	25	NG	6	4
36	26	22	23	6	NG	NCC	21	NG	6	4
37	25	20	25	26	NG	NCC	25	NG	24	4
38	25	27	24	12	NG	NCC	30	NG	30	0.5
39	26	20	26	19	NG	NCC	26	NG	28	8
40	26	20	20	28	NG	NCC	19	NG	25	2
41	24	17	21	17	NG	NCC	20	NG	25	2
42	25	20	26	18	NG	NCC	20	NG	25	2
43	25	23	24	26	NG	NCC	21	NG	27	0.5
44	24	21	20	21	NG	NCC	25	NG	26	4
45	32	30	27	30	NG	NCC	25	NG	30	1

46	24	20	21	18	NG	NCC	23	NG	28	4
47	28	21	23	20	NG	NCC	29	NG	27	4
48	25	21	22	19	NG	NCC	25	NG	24	8
49	22	20	21	23	NG	NCC	24	NG	27	4
130*	26	23	21	19	NG	NCC	21	NG	14	4
131**	24	23	9	6	G	NCC	21	G	25	>256
%Total resistance	0	55	3	68	0	0	0	0	19	10

* *E. faecalis* ATCC 29212. ** *E. faecalis* ATCC 51299. G; growth, NG; no growth, NCC; no color change. AMP; ampicillin (10), CIP; ciprofloxacin (5), C; chloramphenicol (30), E; erythromycin (15), GM; gentamicin (500), N; nitrocefin, P; penicillin (10), S; streptomycin (2000), TE; tetracycline (30), VA; vancomycin (256-0.015). Values shaded dark grey indicate resistance and those shaded light grey indicate intermediate resistance according to CLSI (2015), interpretive criteria. Total percent resistance was calculated for enterococci isolated in this study; no. 19-49.
